# Supplementary material for: Cortico-basal ganglia dynamics of global and selective response inhibition in humans
Source: bioRxiv. 2026 Jan 21:2026.01.20.700500. Preprint. [Version 1] doi: 10.64898/2026.01.20.700500 (PMC12871802; doi:10.64898/2026.01.20.700500)
Supplement: 1 [file NIHPP2026.01.20.700500V1-supplement-1.pdf]

bioRxiv preprint doi: https://doi.org/10.64898/2026.01.20.700500; this version posted January 21, 2026. The copyright holder for this preprint (which was not certified by peer review) is the author/funder, who has granted bioRxiv a license to display the preprint in perpetuity. It is made available under aCC-BY 4.0 International license.

Supplementary Materials

#	Duration (years)	Hoehn-Yahr	UPDRS III	LEDD (mg/day)	MoCA
1	20	3	29	813	>19
2	22	4	33	625	>19
3	13	2	N/A	1750	>19
4	13	2	31	375	>19
5	10	3	N/A	500	>19
6	16	2	48	1225	24
7	10	2	24	250	21
8	8	2	15	660	23
9	8	2	33	731.25	25
10	6	2	45	750	25
11	4	3	35	375	26
12	7	2	53	2000	26
13	4	2	23	250	24
14	3	2	15	0	26
15	8	2	26	375	25
16	11	2	30	0	28

Supplementary Table 1. *Clinical patient characteristics. Duration = Years since initial PD diagnosis. Hoehn-Yahr stage and Unified Parkinson's disease rating scale (UPDRS) Subscale 3 scores quantified ON DBS. LEDD = Levodopa equivalent daily dose in milligrams. Exact Montreal Cognitive Assessment (MoCA) scores were not logged for the first five participants but were above 19.*
